# The CARE (Curiosity, Attentiveness, Respect and Responsiveness, and Embodiment) Model: Operationalizing Cultural Humility in the Conduct of Clinical Research

**DOI:** 10.3390/medicina59112021

**Published:** 2023-11-17

**Authors:** Sana Loue, Timothy Nicholas

**Affiliations:** School of Medicine, Case Western Reserve University, Cleveland, OH 44106, USA; timothy.nicholas@case.edu

**Keywords:** cultural competence, cultural humility, clinical research

## Abstract

Cultural competence training has been criticized for reinforcing existing stereotypes, ignoring intersectionality and inadvertently marginalizing some individuals and groups. In contrast, cultural humility offers the possibility of transformational learning, requiring individuals to pursue a lifelong course of self-examination. This approach makes authentic engagement with others possible. We review the premises underlying cultural competence and cultural humility, as well as proposed models for the integration of cultural humility into the clinical context. We propose a new model for the integration of cultural humility into clinical research: CARE, signifying Curiosity, Attentiveness, Respect and Responsiveness, and Embodiment. We conclude that the concept of cultural humility can be integrated into the conduct of clinical research.

## 1. Introduction

The concept of cultural competence first gained prominence in the United States during the 1980s as an approach to addressing diversity and inequalities [1,2]. It was seen as a potential strategy that could be used to bridge differences that existed between middle-class, often white biomedical clinicians and their patients, whose language and experiences frequently differed from those of their clinicians [3]. Cultural competence pedagogies often assumed that difficulties bridging such gaps were attributable to a lack of knowledge on the part of the clinicians and that these gaps could be remedied through the provision of information about other cultural groups [4]. As such, cultural competence was seen as a tool that would permit an individual or system to better address the needs of their patients or clients who were perceived to be different in some way.

Early definitions of cultural competence tended to view cultures as static and monolithic, reifying and essentializing groups [1,2,5,6,7] rather than recognizing that culture is actively produced through a social process [8]. More recent definitions focus not only on knowledge, but also on attitudes and behaviors [9,10]. This tripartite approach has been criticized as both reductionistic and as reproducing the power dynamic in which the provider is assumed to be the holder of knowledge [1,4].

Later scholarship has focused on various concepts seeking to bridge barriers in communication and understanding between individuals: cultural sensitivity, intercultural communication, and cultural humility [11,12,13]. We focus here on the concept of cultural humility, its key components, and its use in the context of clinical encounters. Finally, we propose a new model that permits the integration of cultural humility into the conduct of clinical research.

## 2. Cultural Humility

Unlike cultural competence which is encounter-based, cultural humility is relationship-based. Tervalon and Murray-Garcia, the originators of the concept, defined it as follows:

It is a process that requires humility as individuals continually engage in self-reflection and self-critique as lifelong learners and reflective practitioners. It is a process that requires humility in how physicians bring into check the power imbalances that exist in the dynamics of physician-patient communication by using patient-focused interviewing and care. And it is a process that requires humility to develop and maintain mutually respectful and dynamic partnerships with communities on behalf of individual patients and communities in the context of community-based clinical and advocacy training models [13] (p. 118).

Scholars have made efforts to identify the key elements of cultural humility. Masters and colleagues suggest that the concept comprises 5 Rs: reflection, respect, regard, relevance, and resiliency [14]. Others have identified active listening, collaboration, egolessness, flexibility, openness, self-awareness, self-evaluation, and self-questioning as important components of the concept [15] (p. 108).

Several models have extended the concept of cultural humility in the clinical context. Grauf-Grounds and Rivera developed the ORCA model: openness, respect, curiosity, and accountability [16]. In this model, respect refers to a sense of self-esteem, honor, and awe for those who are different. Curiosity encompasses the capacity to wonder about and reflect on the context of people’s lives.

Chang and colleagues proposed the QIAN (humbleness) curriculum for engaging with Chinese patients [17]. The model comprises four elements: self-Questioning and critique, bidirectional cultural Immersion, mutually Active listening, and flexible Negotiation. Self-questioning refers to curiosity about oneself and one’s assumptions, as well as curiosity about one’s patients. The element of immersion requires that the clinician approaches each encounter as a cross-cultural exercise. Active listening requires attentiveness to what the patient is saying. Negotiation requires a willingness to understand each other’s belief system and requires efforts to accommodate both and to identify acceptable alternatives when necessary.

Few scholars have discussed the integration of cultural humility into research involving human participants [18]. We suggest that while both the ORCA and the QIAN models may serve as potential frameworks for use in the clinical setting, they fail to address multiple dimensions of the research context. As an example, curiosity in the ORCA model refers to curiosity about the other. However, it is critical in the research context that curiosity be directed as well towards the self: What are the researcher’s motives for pursuing this research question and with this population? Does the researcher have a conflict of interest or bias that is driving their decisions or actions, perhaps to the detriment of the research participants? The QIAN model is potentially useful with populations in addition to Chinese patients. In the context of research, however, negotiation is less flexible at an individual level, in that a research protocol must necessarily be designed to be applicable to all study participants. We propose here a further extension of cultural humility to clinical research through the CARE model: Curiosity, Attentiveness, Respect and Responsiveness, and Embodiment.

## 3. CARE: A Model for the Integration of Cultural Humility into Clinical Research

### 3.1. Curiosity

Curiosity is “the desire to seek information to address knowledge gaps resulting from uncertainty or ambiguity” [19]. Scientific thinking intentionally seeks knowledge, asks questions, makes observations and inferences, and observes patterns [20]. Something is unknown, ambiguous; curiosity motivates the seeker to learn more. Curiosity fosters a sense of wonder; the more one knows, the less one takes for granted [21]. Curiosity is critical for the development of scientific thinking and drives the motivation to pursue scientific questions [19]. Curiosity as conceived within the framework of cultural humility concerns not only the research question and the research participants, but the researcher and research team members as well. It is incumbent upon the researcher to continually explore their motivations for pursuing a specific research question and their selection of participants. Researchers might ask themselves what their pre-existing assumptions or biases might be with respect to the participant population; whether their pursuit of the specific scientific question with a specific population is driven by additional potentially problematic motivations, such as financial recompense; and what values and priorities they may bring to the research that may differ from those of the research participants and/or other stakeholders.

### 3.2. Attentiveness

Attentiveness has been variously defined as “the state of being awake, alert and actively paying attention to a stimulus” [22]. The Oxford Learner Dictionary refers to attentiveness as “the quality of listening or watching carefully with interest” [23]. The French philosopher Simone Weil referred to attention as “the rarest and purest form of generosity” [24]. Attentiveness requires that the observer not only abandon stereotypes that they may be bringing to the interaction and the false sense of security that often comes with reliance on stereotypes, but also focus on multiple dimensions and on the whole simultaneously [18,25].

Attentiveness requires active listening and may be communicated through nonverbal or verbal behaviors. Nonverbal behaviors include head nods, eye contact, and a forward body lean that reflects a closer psychological distance between individuals [26]. Such behaviors communicate approach, in contrast to avoidance and signal involvement, attentiveness, and awareness [27,28]. Verbal behaviors include paraphrasing what the research participant has said, reflecting back their feelings, checking one’s assumptions, and asking questions of the research participant.

### 3.3. Respect and Responsiveness

Respect signifies a willingness to engage as equals, to interact as I–You rather than I–It, in which the other individual is addressed as an object [29]. This engagement as equals minimizes the power imbalance that exists between the researcher and the research participant [30]. The I–You relation does not refer to ordinary conversations, but rather signifies “one whole being encountering or confronting another whole being” [31] (p. 209). Respect requires that one listens to understand, rather than listening with the intent to respond from one’s imagination or memory. According to Buber,

Genuine conversation, and therefore every actual fulfillment and relation between men, means acceptance of otherness … Everything depends, as far as human life is concerned, on whether each thinks of the other as the one he is, whether each, that is, with all his desire to influence the other, nevertheless unreservedly accepts and confirms him in his being this man and in his being made in this particular way [32] (p. 59).

Buber continued,

Unlike the realm of experience, the I-You is unmediated: Nothing conceptually intervenes between the I and You, no prior knowledge and no imagination, and memory itself is changed as it plunges from particularity to wholeness. No purpose intervenes between I and You, no greed and no anticipation, and longing itself is changed as it plunges from the dream into appearance. Every means is an obstacle. Only where all means have disintegrated encounters occurs [29] (pp. 62–63).

The philosopher Hermann Cohen recognized the intertwined existence of I and Thou in the establishment of a relationship:

that *I* cannot conceive *I* without conceiving *Thee*. So, in self-consciousness, the Other has transformed into Thou in a duality with *I*. So as far as self-consciousness means the unity of will, it has to create the union of *I* and *Thou* [33] (p. 514).

Accordingly, Cohen conceived of the other not as a Nebenmesch, meaning a “man next to me,” but rather as a Mitmensch, a “man with me” [34] (pp. 20–21). Cohen suggested that it is an

ethical action which turns the other man into Thou, the *Mitmensch*, it is the effective action for eliminating his suffering … In this correlation man constitutes the Other as Thou, but at the same time, constitutes himself as Thou for the Other: thus compassion is not a reflexive feeling of a constituted Ego who, by means of reduction of the Other to himself returns to himself, but it is ethical action, while taking up the suffering Other as *Mitmensch* constitutes himself at the same time in correlation … This process, then, is not only recognizing the similarity of the Other, but real production of man by means of an ideal process discovering in the diversity of *Nebenmensch* the similarity of *Mitmensch* inasmuch as he is simply *Mensch* [35] (pp. 137–138).

Cohen derived his approach from what he conceived of as the ethical imperative to direct love outwardly to the “other.” In so arguing, he relied on Leviticus 19: 33–34, which commands:

When a stranger resides with you in your land, you shall not wrong him. The stranger who resides with you shall be to you as one of your citizens; you shall love him as yourself, for you were strangers in the land of Egypt: I the LORD am your God [36].

The Hebrew text uses the word ger, generally understood to encompass both shorter-term travelers and long-term sojourners and resident aliens as well [37]. The ger was

a man of another tribe or district, who, coming to sojourn in a place where he was not strengthened by the presence of his own kin, put himself under the protection of a clan or powerful chief. From the earliest times of Semitic life the lawlessness of the desert has been tempered by the principle that the guest is inviolable. A man is safe in the midst of his enemies as soon as he enters a tent or touches a rope. To harm a guest or to refuse him hospitality, is an offense against honour which covers the perpetrator with indelible shame … The obligation thus constituted is one of honour, and not enforced by human sanction except public opinion, for if the stranger is wronged he has no kinsmen to fight for him [38] (p. 76).

The situation of a man of another tribe entering an unfamiliar and potentially hostile community is analogous to that of an individual entering the relatively unfamiliar research environment as a study participant. Is he safe? Has the researcher developed and instituted adequate and sufficient protections to safeguard the individual from potential harm and/or to care for him should unforeseen harm occur? Does the researcher regard the participant as “a man next to him” or as “a man with him,” recognizing that the research could not be carried out without the participants, and that participants are truly partners in the research enterprise.

It is the recognition of both similarity and difference and the acknowledgement and acceptance of the Other as “man with me” that facilitates responsiveness to the Other. Responsiveness is grounded in the ongoing, shifting confluence of knowledge and understanding. Responsiveness requires an awareness of oneself and one’s triggers, attunement to the other, and response rather than reaction [39].

### 3.4. Embodiment

Embodiment requires attunement to person, self, and context. Accordingly,

[i]mplicit within the concept of embodiment is a sense of dynamism or constantly shifting meanings and understandings. Embodiment is experienced within particular historical, cultural, political and societal frames and these experiences are also shaped by gender and race [40] (p. 41).

Embodiment is inherently relational, involving relationships between persons and between persons and their environments [41]. Embodiment involves bodily resonance; our neural systems recreate what others do and feel, such that people model others’ behavior or mental states as intentional experiences, that is, as “embodied simulation” [42]. There is an intuitive understanding of others that occurs in ongoing interactions, often on a pre-reflective level. A process of mutual modifications of bodily and emotional states may take place as a result of bodily presence [43]. In discussing what transpires between persons engaged in an authentic exchange, Buber observed that

What is essential does not take place in each of the participants or in a neutral world which includes the two and all other things; but it takes place between them in the most precise sense, as it were in a dimension which is accessible only to them both [44] (pp. 203–204). *s*

The development of embodiment requires close attention to the body as well as individual assessment: is what I am doing congruent with what I am feeling? Such a focus has been found to be associated with higher levels of empathy [45].

### 3.5. Applying the CARE Model

The Cambodia Pre-exposure Prophylaxis study offers an opportunity to explore how the integration of cultural humility into research may have facilitated a different outcome.

This first large-scale clinical trial in Cambodia, begun in 2003, was conducted by the University of California San Francisco and the University of New South Wales in collaboration with the Cambodian National Center for HIV, AIDS, Dermatology, and Sexually Transmitted Infections (NCHADS) and was funded by the U.S. National Institutes of Health and Family Health International, which had received a grant from the Bill and Melinda Gates Foundation [46]. In that same year, then-President George W. Bush announced the creation of the President’s Emergency Plan for AIDS Relief (PEPFAR) [47].

The trial was designed to test the efficacy and safety of the antiretroviral drug tenovir disoproxil fumarate to prevent HIV: Is one pill per day safe for HIV-negative people? Does the drug prevent HIV infection? The trial was designed as a double-blind randomized controlled trial among 960 sex workers in Phnom Penh. Participants were to receive tenofovir or a placebo [46]. All participants were to receive USD 5.00 per visit, free condoms, HIV testing and counseling, and testing and treatment for sexually transmitted infections. Participants were to be monitored for signs of drug toxicity, pregnancy, risk behaviors, and HIV seroconversion.

The trial was canceled in 2004 following protests from organizations representing sex workers, including the Women’s Network for Unity and Womyn’s Agenda for Change and after the country’s Prime Minister spoke out against the research [46,48]. The organizations were concerned that participants’ rights were not adequately protected and that there had been no guarantee of access to the drug after the trial if it were found to be effective. The organizations demanded that the researchers provide long-term health care in the event of adverse drug effects and 30 years of post-trial health insurance.

In response to these criticisms, the researchers insisted that they had adhered to international bioethical standards [46]. They had received approval from the ethics committees of both collaborating academic centers, the Cambodian Ministry of Health National Ethics Committee had approved an early draft of the protocol, and the U.S. National Institutes of Health had extensively reviewed the protocol. The Cambodian Ministry of Health established an external advisory group and the NCHADS established a community advisory board [46]. Neither of these two advisory groups had adequate time to develop operating procedures prior to the study discontinuation. The researchers argued that 30 years of health insurance would constitute an unethical inducement, but agreed to provide two years of post-trial care and access to tenofovir at a reduced cost. Participants who seroconverted during the course of the trial would be referred to the NCHADS HIV clinic for assessment and care [46].

The trial has been characterized as an example of a situation that required “postcolonial bioethics” [49]. Postcolonial bioethics demands consideration of a history of oppression and the avoidance of actions that are performative [50]. It is

shorthand for bioethics at a conjuncture of experiences of decolonization and struggles over sovereignty and global circulatory capacities of human rights, biomedical research ethics, and medical interventions themselves … The concept of postcolonial bioethics captures how global health science is explicitly about relations, relations intertwined with other formations of vulnerability and responsibility” [49] (p. 238).

The researchers focused on their adherence to ethical principles, conceiving them as universal. However, those opposing the trial focused on the unequal relations between the funders, regulatory bodies, and the participant community. The trial was perceived by its opponents as a binary undertaking: foreign investigators acting on Cambodian sex workers, with Cambodian collaborators essentially unseen. The implication was that Cambodians were to be exploited for the benefit of non-Cambodians.

We can ask whether the integration of cultural humility through the CARE model might have made a difference in the fate of the tenofovir trial. There are various unknowns here: whether the investigators and/or the U.S.-based ethics review committees held implicit assumptions about sex workers and/or Cambodians and the effect of the refusal by Womyn’s Agenda for Change to sign the 2003 “anti-prostitution” pledge required by the U.S. government for the receipt of funds under PEPFAR.

Nevertheless, the integration of cultural humility and, specifically, reliance on the CARE model, may have yielded increased engagement and collaboration with the relevant communities and enhanced the investigators’ and funders’ understanding of and sensitivity to the local context. Greater curiosity about the history of Cambodia, the societal and economic status of the sex workers, and the women’s various motivations for engaging in sex work may have prompted the researchers to examine in greater depth the context in which they were to conduct the research. They might then have better understood the demand for a safety net for the sex workers and their families if adverse events were to occur, as well as the impact of the country’s colonial history on community perceptions and understandings of the trial. The integration of curiosity, respect, and responsiveness might have prompted the investigators to engage with relevant stakeholders, including organizations representing the sex workers, earlier in the research process and to negotiate issues of safety and compensation with them. The researchers might have then understood that the sex workers did not want to be treated as “individuals next to me,” but rather as “individuals with me,” as equal partners relating in the context of research.

The absence of cultural humility is similarly reflected in other studies, albeit involving different research questions and populations. Researchers conducting studies in post-apartheid South Africa between 1997 and 2004 concluded from their survey research with non-White South African adolescents and young adults that, contrary to then-prevailing beliefs, they were not “immune” to eating disorder pathology [51,52,53,54,55,56]. On the basis of such reports, previous understandings of anorexia and bulimia nervosa as “culture bound” due to Western ideals of female thinness were expanded and relabeled as “culture reactive” disorders, i.e., affecting individuals in societies that were experiencing “culture change” [57,58].

However, later research conducted by Le Grange and colleagues utilized both eating disorder measures and structured interviews to ascertain the existence of eating disorder pathologies in a similar population [59]. Some respondents reported a preoccupation with food and eating or vomiting following the ingestion of food. However, they variously explained these behaviors as the result of extreme poverty and a consequent preoccupation with food due to chronic hunger or the need to ingest the only available food but then to engage in purging because the specific food was prohibited by religious or cultural beliefs. The authors suggested, on the basis of the interview responses, that the concept of eating disorders had been “uncritically transferred across cultures” [59] (p. 448). This apparent misclassification of participants reflects more than an uncritical application of a diagnostic concept and associated measures. Rather, it evidences the absence of curiosity about the specific context of the study and the study participants and the researchers’ non-responsiveness to that context.

Yet another study illustrates the harm that may result to an individual participant due to a researcher’s failure to engage in self-reflection, including an examination of their own biases and motivations, as well as a lack of humility, respect, and responsiveness to the context in which a research participant lives. Although the following events occurred in 2003 and 2004, the underlying circumstances that gave rise to participant harm remained unclear until 2015.

Dan Markingson, then 26 years old, experienced his first psychotic break in 2003. On 12 November 2003, he threatened to kill his mother. In response, his mother called the police, who transported Markingson to the Regions Medical Center in St. Paul, Minnesota [60]. Due to a lack of psychiatric beds, he was transferred to Fairview University Hospital, a teaching hospital for the University of Minnesota Academic Health Center [61]. There, Dr. Stephen Olson, an associate professor of psychiatry, treated Markingson with resperidone, a medication frequently prescribed to patients with schizophrenia or bipolar disorder. Olson determined that Markingson was psychotic and dangerous and lacked the necessary capacity to make medical care decisions for himself, circumstances that supported the involuntary administration of antipsychotic drugs under Minnesota state law. A court-appointed psychologist examined Markingson on November 19, 2003. He concluded that Markingson displayed a “gross impairment of judgment, behavior, capacity to recognize reality, [or] capacity to reason or understand” [60].

On 20 November 2003, Olson requested a stay of commitment for Markingson. This would allow Markingson to avoid involuntary commitment to a mental hospital as long as he agreed to comply with their psychiatrist-recommended treatment program. The court granted such a stay for a period of six months [61]. However, rather than fashioning a treatment program, Olson enrolled Markingson in a clinical trial that he led. The trial, known as the CAFÉ Trial (Comparison of Atypicals in First Episode), was designed to evaluate three antipsychotic drugs for the treatment of first episode schizophrenia: quetiapine (Seroquel), olanzapine (Zyprexa), and risperidone (Risperdal). The trial was funded by AstraZeneca, the manufacturer of olanzapine.

The CAFÉ study was designed to be a double-blind trial, with random assignment to a specific arm to be made by computer. Participants were not permitted to be taken off their assigned drug or switched to another drug if the one to which they were assigned was not working. The trial also restricted the use of medications that could manage the side effects or symptoms of depression or anxiety. The study coordinator, Jean Kenney, a social worker by training, had Markingson sign an informed consent form on 21 November 2003. The form was signed without either Markingson’s mother or a study advocate present, although Olson had represented to the university’s institutional review board that all study participants would have an advocate [60]. It is unclear to what extent Markingson did or did not understand the risks related to study participation or whether he believed that the stay of commitment could be revoked and he could be involuntarily committed if he did not enroll in or if he withdrew from the study. Olson continued to serve as Markingson’s psychiatrist and as the lead of the CAFÉ study.

At the time of Markingson’s trial enrollment, Olson and his research team were under pressure from AstraZeneca to address their slow rate of participant recruitment or face the possibility of having the study site shut down. The closure of the study site would result in a loss of the payment from AstraZeneca for participants’ enrollment and completion of study visits. Although these funds did not directly impact the salaries of either Olson or Kenney, the university’s budget depended upon faculty generation of external funding [60].

Markingson was transferred to a halfway house on 8 December 2003. He was required at that time to sign a statement indicating his understanding that he could be involuntarily committed if he did not continue taking his prescribed medication and adhering to his CAFÉ study appointments.

Markingson’s mother noted her son’s continuing mental deterioration at the halfway house and communicated her concerns on various occasions to Olson, to Kenney, and to Charles Schulz, the chair of the psychiatry department and the co-investigator of the CAFÉ study. Schulz responded on only one occasion, commenting that “it was not clear to [him] how [she] thought the study team should deal with this issue” [61]. Staff reports from the group home reflected similar observations of Markingson: increased inattentiveness, an increasingly disheveled appearance, and a glaze in his eyes. On 8 May 2004, Markingson killed himself at the group home by slashing his neck and abdomen with a box cutter, nearly decapitating himself [60,61]. A later toxicology report found that the only drug in his system at the time of his death was the study drug quetiapine [62].

The Markingson case reflects the absence of investigator curiosity with respect to both his own motivations and Markingson’s health status. Olson was neither attentive nor attuned; there is no indication that he engaged in active listening to Markingson or that he was attuned to self, to Markingson, or to Markingson’s living context. Olson clearly had a conflict of interest here in that he served as both the lead on the study and as Markingson’s psychiatrist. Curiosity about the self would have required that he acknowledge the existence of the conflict; respect would have required that he actually manage the conflict in such a way as to mitigate any potential harm to Markingson that could result.

## 4. Discussion

We have suggested that cultural humility can be integrated into research involving human participants through reliance on the CARE model, which comprises the elements of Curiosity, Attentiveness, Respect and Responsiveness, and Embodiment. The success of such efforts and of our model necessarily rests, at least in part, on researchers’ ability to understand and to integrate the concept of cultural humility.

Scholars have suggested a wide range of approaches to facilitate health care providers’ development of cultural humility at various stages of their careers, several of which appear to yield more promising results than others. These have included panel discussions, interactions with simulated patients, home visits, book and video discussions, relationship-centered interview training, engagement with the humanities [63], reflective journaling, modeling cultural humility [64,65], and mindfulness training [18]. Reflective journaling and mindfulness meditation have, in particular, been found to promote the development of various elements of cultural humility. Reflective journaling has been found to enhance self-awareness and self-understanding [66,67]. Mindfulness meditation, which is “characterized by a present-oriented consciousness in which individuals focus on moment-to-moment experiences rather than thinking about the past or fantasizing about the future” [68] (p. 311), may enhance an individual’s ability to recognize their values and needs [64], regulate emotion [69,70,71], and decrease automatic thought and behavior patterns so that they may respond more flexibly and with self-determined behavior [72].

One of the more promising approaches for developing cultural humility in the context of research may be that suggested by Murray and colleagues [73]. They suggest organizing writing accountability groups, holding DEI (diversity, equity, inclusion) Fridays for discussions of biases and group members’ values and cultures, holding special sessions to focus on the development of skills that comprise cultural humility, and practicing reverse mentoring, whereby the mentor also learns from their mentees. Their approach, developed specifically for use in the context of research and research training, comprises activities that can easily be integrated into lunch hours and team meetings.

How and whether ethics review committees should assess the CARE model of cultural humility, or cultural humility at all, within research teams as they review research proposals remain open questions. Ethics review committees often seek cultural consultation when reviewing proposed protocols that involve marginalized populations and populations located outside of the U.S. In the view of the authors, this is a necessary component of the ethics review, but is not sufficient to ensure that research team members approach their participants with CARE.

We suggest that ethics review committee members consider the following questions as they review protocols in order to evaluate the extent to which cultural humility has been integrated into the research design and the functioning of the research team.

*Curiosity:*

- How and to what extent has the research team familiarized itself with the social, cultural, economic, and historical context of the proposed participants/participant community?
- How and to what extent do research procedures facilitate an examination of team members’ biases and values and their potential impact on the research participants and the course of the research?

*Attentiveness*

What stereotypes or assumptions about the study site and/or participants do the researchers bring to the endeavor?
How have individual research team members who will have contact with participants or analyze data been trained to interact with participants and/or examine how their assumptions may impact their data interpretation?

*Respect and Responsiveness*

- To what extent, in what way, and for how long has the research team engaged with the prospective participant community?
- What mechanisms have been put in place to reduce the power imbalance between the researcher and research participants and/or the participant community?
- Are the research protections and levels of participant remuneration responsive to participants’ concerns, as well as federal regulations and international guidelines?
- Are representatives of the participant community consulted for their input? In other words, do they have a seat at the table?

*Embodiment*

- Have the researchers considered the potential implications for participants as a result of their contact with team members, e.g., if there may be political or social repercussions from contact with foreigners? How will they manage this potential?
- Have the researchers developed adequate measures to protect the identity of the participants?
- Have the members of the research team designed the study in such a way as to be sensitive to local customs and mores relating to personal space and interpersonal communication?

Additional research is necessary to evaluate the acceptability and usefulness of our model. Additionally, the various measures that exist to assess cultural humility have been developed for use in the clinical context [74,75,76,77]. Whether one or more of them can be utilized in the context of research to assess research team members’ levels of cultural humility remains unknown.

## 5. Conclusions

We suggest that our proposed CARE model of cultural humility, comprising curiosity, attentiveness, respect, responsibility, and embodiment, can be integrated into the research context. We draw extensively from both Martin Buber and Hermann Cohen, drawing parallels between the relationship between the researcher and research participant, on the one hand, and the individual and the stranger, on the other. We have provided several examples of studies in which cultural humility appeared to be absent: the Cambodia tenofovir study with sex workers, various studies relating to eating disorder symptomatology among South African adolescents and young adults, and the enrollment of Dan Markingson by his treating psychiatrist, while under court order, in a three-arm clinical trial for first episode schizophrenia. These examples illustrate the potential consequences of moving forward in the absence of cultural humility: cessation of the research, development of community distrust of researchers, faulty assumptions underlying the research resulting in faulty conclusions, and harm to the individual participant. We have provided suggestions for ethics review committees that their members may use in reviewing protocols to assess the extent to which the research study design and the research team have integrated cultural humility. Additional research is necessary to assess its acceptability among researchers, to examine how it can best be taught and evaluated, and to determine how the model can best be utilized by ethics review committees as they review proposed protocols.

## Data Availability

Not applicable.
